# Formation and Evaluation of Complete Blood Count Proficiency Testing Program

**DOI:** 10.3390/hematolrep14020012

**Published:** 2022-03-25

**Authors:** Huy Quang Vu, Oanh Hoang Le, Duan Cong Truong, Dung Ngoc Nguyen, Triet Hy Van, Van Thi Kieu Le, Linh Thi Truc Vang

**Affiliations:** 1 Faculty of Nursing and Medical Technology, University of Medicine and Pharmacy at Ho Chi Minh City, Ho Chi Minh City 748010, Vietnam; vanhytriet@gmail.com; 2 Quality Control Center for Medical Laboratory, University of Medicine and Pharmacy at Ho Chi Minh City, Ho Chi Minh City 748010, Vietnam; ltkvan@ump.edu.vn (V.T.K.L.); vangthitruclinh97@gmail.com (L.T.T.V.); 3 Cho Ray Transfusion Blood Center, Cho Ray Hospital, Ho Chi Minh City 748010, Vietnam; lhoanh480@gmail.com; 4 Laboratory Department, Vinmec Times City International Hospital, Hanoi 100000, Vietnam; v.duantc@vinmec.com; 5 Department of Cytology and Histology, National Institute of Hematology and Blood Transfusion, Hanoi 100000, Vietnam; bsdungnihbt0874@gmail.com

**Keywords:** completed blood count, external quality assessment, homogeneity, stability, ISO 13528:2015

## Abstract

**Introduction**: The haematology external quality assessment (EQA) scheme is the most commonly used service of quality assurance. The provision of complete blood count (CBC) materials must meet the quality requirements at a reasonable cost. These requirements are the most significant challenges for EQA organisers in Vietnam. This study’s objective was to evaluate the homogeneity, long-term stability, and peer-group performance of 10-parameter stabilised CBC EQA samples. **Methods**: The CBC EQA material was prepared using the following steps, including (1) adjusting levels of stabilised erythrocyte, leukocyte, and platelet samples, (2) mixing those cells into batches at three levels, and (3) dispensing and storing them at 2–6 °C. A set of 10 and 30 specimens were randomly chosen from each batch to study the homogeneity and long-term stability following ISO 13528:2015. In total, 166 samples at two levels were randomly distributed to 40 participants, which reported 83 automatic cell counters among six automated analyser models in the CBC EQA program. **Results**: The 10-parameter stabilised CBC EQA materials at three levels became homogeneous and stable in 12 weeks when preserved at 2–6 °C. Meanwhile, for five parameters (RBC, Hb, MCH, MCV, and MPV), this process was prolonged for up to 16 weeks in stock condition. In terms of peer-group performance, the CV (%) values increased at the low concentration for almost all parameters, especially in platelet counts. **Conclusions**: The stabilised CBC EQA samples prepared using the partial fixation method with aldehyde and gutaraldehyde in this study meet the ISO 13528:2015 requirements of homogeneity and long-term stability for the CBC EQA scheme. Analytical performance evaluation should categorise participant methods into peer groups.

## 1. Introduction

Complete blood count (CBC), performed using automated haematology analysers, is the most common blood test indicated by worldwide clinical rates. Thus, the CBC External Quality Assessment (EQA) scheme is one of the most crucial tools in the quality assurance of laboratory quality management and is necessary for medical laboratories seeking harmonisation and accreditation according to the international standard ISO 15189 2012 [1,2,3]. A traditional EQA scheme includes the following steps: laboratories receive a set of samples with similar characteristics supplied by the EQA organisation. The next step is to measure one or more of the components in that sample in the same manner as for the patient sample. Finally, the results return to the EQA organisation to be analysed and evaluated by comparing with the assigned values. After that, the EQA reports the assessment of the performance of the laboratory to allow for opportunities for improvement.

The CBC EQA scheme from EQA organisers in Vietnam is mostly based on materials which are imported from foreign suppliers. Thus, it involves quite a high cost for participating laboratories. To achieve the goal of independence in the production of EQA materials, we applied the use method of stabilised whole blood as a CBC EQA material using Aldehyde (based on WHO guidelines [4]) to build a standard procedure for EQA material preparation in large volumes.

This research aimed to evaluate the homogeneity, long-term stability according to ISO/IEC 17043:2010, and peer-group results of stabilised whole blood as an EQA material using 10 parameters, including haemoglobin concentration (Hb), red blood cell (RBC) count, haematocrit (Hct), mean cell volume (MCV), mean cell haemoglobin (MCH), mean cell haemoglobin concentration (MCHC), red cell distribution width (RDW-CV), white blood cell (WBC) count, platelet count (PLT) and mean platelet volume (MPV).

## 2. Materials and Methods

### 2.1. Material Preparation

The blood components were collected from the Cho Ray Transfusion Blood Center. All the blood products were checked and had negative results in terms of HIV, HBV, HCV, syphilis, and malaria; moreover, the blood components were required to have the same ABO blood group and had to be negative in the direct/indirect Coombs test. The criteria used for the selection of blood components was that the time from collection to production was not over 24 h, 72 h and 7 days for leukocyte, platelet, and red blood cell components, respectively [5]. The phosphate-buffered saline (PBS) and cell suspension solutions (CSS) were also prepared following the instructions in [6,7]. Three cell lines of red blood cell, leukocyte, and platelet components were produced separately using the procedures that follow.

RBC: Firstly, red blood cells were distributed into centrifuge tubes, washed twice with PBS solution (2500 rpm in 20 min), and the buffy coat was removed. Then, they were immobilised using 0.0025% glutaraldehyde solution (one volume of red blood cell in nine volumes of immobilising solution), while constantly being shaken for 1 h at room temperature. Next, they were washed three times with PBS solution to remove the glutaraldehyde solution, diluted with CSS solution and stored at 2–6 °C for 24 h. Finally, the CSS solution was replaced via centrifugation (2500 rpm in 20 min), the supernatant was removed, and new CSS solution was added and stored at 2–6 °C until use.

WBC: Separation was performed on platelet-rich plasma by diluting leukocyte-rich product with PBS solution (ratio 1:1), which was centrifuged at 800 rpm for 10 min, to eliminate the supernatant. Then, it was centrifuged at 2000 rpm for 10 min to collect the buffy coat. Next, erythrocytes were lysed by adding the lysate (ratio 1:5) for 10 min. Leukocytes were separated by centrifugation at 2500 rpm for 5 min; then, the supernatant was removed, and the leukocytes were washed (two times) with PBS solution. The leukocytes were immobilised using 10% formaldehyde (ratio: 1:10) for 20 min at 50–60 °C; they were gently mixed, and then, the temperature was reduced to 20–25 °C. Next, they were washed three times (and centrifuged at 2500 rpm for 5 min) with PBS solution to remove the formaldehyde solution. Finally, the white blood cells were suspended in CSS solution and stored at 2–6 °C.

PLT: Platelets were distributed into 15 mL Falcon tubes and using a 0.16% formaldehyde solution (ratio of 1: 1), they were stabilised. Then, they were mixed gently using a shaker for 15 min at room temperature. Then they were washed three times with PBS via centrifugation (1500 rpm in 40 min), and the supernatant was removed. Finally, diluted platelets were placed in CSS solution, and stored at 2–6 °C.

Stabilised whole blood samples for use as automated counting EQA assay materials were prepared by pooling suspensions of RBC, WBC, and PLT with the same ABO blood groups as suitable ratios of each component to obtain the desired levels. Those blood cells were previously partially fixed with formaldehyde and glutaraldehyde, and then they were suspended in an isotonic solution, pH = 7.4, osmolality = 315 mOsm/L, including antibiotics to maintain sterilisation. Whole-blood specimen pools were dispensed, bottled, and stored at 2–6 °C for up to 4 months. The total of 150 2mL aliquots were prepared from each pool, and three level ranges were chosen in this study to make three batches [8,9]:

- Level 1: RBC (1.5–3.0 10^12^/L), WBC (2–4 10^9^/L), and PLT (40–150 10^9^/L)
- Level 2: RBC (3.5–4.5 × 10^12^/L), WBC (5–9 × 10^9^/L), and PLT (150–350 × 10^9^/L)
- Level 3: RBC (4.5–6.0 × 10^12^/L), WBC (9–30 × 10^9^/L), and PLT (400–700 × 10^9^/L)

### 2.2. Homogeneity and Stability

The homogeneity and stability of whole-blood samples for use as EQA assay materials were determined according to ISO/IEC 17043:2010 [10] and ISO 13528:2015 [11]. Regarding the homogeneity, 10 specimens selected from each pool were analysed in duplicate to determine complete blood count (CBC) parameters immediately after bottling. For stability, two samples were chosen randomly from each batch and analysed in duplicate every week for 4 months. Within- and between-batch analysis was used to confirm the stability and the homogeneity of the batch for 10 parameters using a Sysmex XE-5000 analyser (Sysmex, Kobe, Japan).

### 2.3. Specimen Distribution

**Figure 1:**
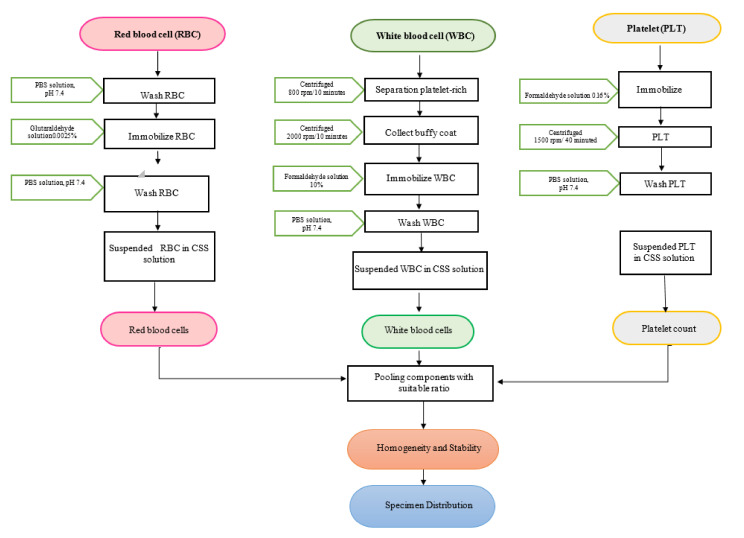
Flow chart of the research process.

This study included data from 166 specimen pools from two batches at level 1 and level 3, which were randomly distributed to 40 participants in the CBC EQA scheme. A total of 83 automatic cell counters among six models were registered, including Sysmex XN-1000 series, Beckman Coulter D × H 600/800/900 series, Mindray BC 1000/2000/3000 series, Nihon KohdenCelltac Alpha, Siemens/Bayer Advia 120/2120, and Abbott Cell-Dyn Ruby by first class post within Vietnam. Two CBC EQA samples were delivered to participants in 2–6°C conditions. The transit time varied from 1 to 2 days on average, and the closing date for the return of results was 10 days from the distribution date. The whole procedure was depicted in Figure 1.

### 2.4. Instrument Grouping

EQA participants were grouped into six instrument types, as mentioned for the analysis, according to analyser method. The minimum target number of participants in each group was 12 to eliminate outliers, according to ISO 13528:2015 [11]. To counting red blood cells, white blood cell, and platelet counts, two methods were available in those six analyser models: optical and impedance.

### 2.5. Statistical Analysis

Data were entered and analysed using Microsoft Excel 2019. Outliers were identified and removed using Cochran’s test. The assigned values (the mean of the values after outliers were removed), the uncertainty of measurement of the assigned value (Um), and the coefficient of variation (CV) values were produced for individual instrument group data.

- Assigned value ($\bar{X}$ or mean): determined by the consensus of the participating laboratory; the mean when the data are normally distributed or median when the data are not properly distributed.
- SD: standard deviation

In which:

(1) $$SD=\sqrt{\sum_{i=1}^{n}\frac{{\left(x_{i}-\bar{X}\right)}^{2}}{n-1}}$$

n: number of participating laboratories that were involved in the evaluation for each model of haematology analysers

*x_i_*: results of participating laboratories in testing *i* (*i* = 1, 2, 3,..., *n*)

CV (%): coefficient of variation,

(2) $$CV\;\left(\%\right)=\frac{SD}{\bar{X}}\;x\;100$$

U_m_: uncertainly of measurement, presenting the confidence of assigned value. $Um=1,25\times \frac{SD}{\sqrt{N}}$*,* in which N is the number of selected results for mean calculation after the elimination of outliers.

Assigned value ($\bar{X}$) is considered as enough confidence when: *U_m_* ≤ 0.3 *ϭ_pt_*

In which

*ϭ_p_*_t_ is the allowed standard deviation

*ϭ_pt_* = TEa × $\bar{X}$, (TEa: total allowable error goal)

## 3. Results

### 3.1. Homogeneity

Table 1 shows the specimen homogeneity during the dispensing process. According to ISO 13528:2015 [11], CBC EQA control materials are considered to be adequately homogeneous if s_s_ ≤ 0.3 ϭ_pt_. It can be observed that the differences between the sample standard deviations (s_s_) of 10 parameters, measured in duplicate from each specimen at three levels, were mostly smaller than 0.3 ϭ_pt_. Thus, this CBC EQA assay material was homogeneous and adequate use in this study.

### 3.2. Stability

Table 2 demonstrates specimen stability over 4 months’ storage. According to ISO 13528:2015, CBC EQA materials are adequately stable if $\left|{\underline{y}}_{t}-{\underline{y}}_{1}\right|\leq$*0.3* *ϭ_pt_,* t: time of storage. Hence, it can conspicuously be seen that all ten parameters at three levels were stable for 12 weeks when stored at 2–6 °C. Notably, the stability of five parameters (RBC, Hb, MCH, MCV, and MPV) was extended and prolonged for up to 16 weeks. In addition, WBC, and PLT values at all levels tended to decrease gradually with the time of storage, and their stability was maintained for up to 13, and 14 weeks, respectively. Otherwise, the RDW-CV percentage at all levels tended to increase continuously and became unstable from the 13^th^ week of the 16-week follow-up. Additionally, the Hct and MCHC stability changed according to the concentration ranges. In particular, from the low to the high level, the stable period of Hct was reduced from 13 weeks (level 1and 2) to 12 weeks (level 3) which caused to the MCHC stability to rise from 13 weeks (level 1) to 16 weeks (level 2 and 3).

### 3.3. Peer-Group Evaluation

The results of 83 analyser models from six instrument groups were compared in this study (Table 3). From the 166 EQA assay material pools sent to participants, 75 and 78 individual results at level 3 and level 1, respectively, were used after removing outliers to calculate the mean, Um, and CV for each parameter. The average result of each peer group (mean) was used as the assigned value [12] to compare performance to different instruments and analytical methods. As reflected in Table 3, the mean values of RBC, WBC, and PLT at both levels were within the desired ranges from the sample preparation protocol. In addition, the standard uncertainty (Um) of the assigned value did not exceed the upper limit (0.3 ϭ_pt_) [11] in all parameters except RDW-CV (Table 4). Thus, the assigned value was reliable, and the uncertainty was negligible for most of the measurements. On the other hand, the uncertainty (Um) of RDW-CV should be combined with the standard deviation for performance assessment.

As can be seen in Table 5, the all-methods CV value of seven red blood cell parameters (RBC, Hb, Hct, MCH, MCV, MCHC, and RDW) tended to mildly decline at level 3 versus level 1, mostly ranging under 3 % for all peer groups, except Mindray BC 1000/2000/3000 series (CV of MCHC, 3.6%), and Nihon KohdenCeltac Alpha (CV of Hct, 3.1%; CV of RDW-CV, 4.8%).

The CV increased as the platelet count (PLT) decreased (Table 4), ranging from an all-methods value of about 5.2% (level 1) to about 2% (level 3). This same tendency was also noticed for all analyser model groups, with CV values two to four times greater at counts of 94.6 to 122.4 × 10^9^/L (CV range, 3–8%) compared with counts of 526.7–569.0 × 10^9^/L (CV range, 1–3%). Similarly, the CV of total leukocyte count (WBC) was slightly higher at level 1 than level 3, ranging from an all-methods value of about 2.8% to about 2.5%, respectively. Additionally, the CV range of WBC at the count of 2.2 to 2.6 × 10^9^/L (CV range, 2–4%) was more extensive than that at the count of 9.5 to 11.0 × 10^9^/L (CV range, 1–3%).

Moreover, from Table 5, it can be seen that the CV range of MPV was the widest between peer groups at both level 1 (1–8%) and level 3 (2–9%) because the CV values of Abbott Cell-Dyn Ruby and Nihon Kohden Celtac Alpha were three to five times than other groups’ values, at counts of 5.8 to 6.4 fL (CV range, 6.1–6.9%) and counts of 8.1–8.4 fL (CV range, 7.6–8.3%).

## 4. Discussion

A human whole-blood sample is the ideal control material; however, it cannot be routinely applied because of limited stability especially in WBC and PLT parameters. A comparison between clinical samples and quality control materials was made in the research of Vidali M. [13]. The stabilised CBC specimen pools for use as CBC EQA assay materials prepared by being partially fixed with aldehyde in this study were homogeneous and stable for at least 12 weeks for 10 parameters at three levels. This preparation method was based on WHO guidelines [4] and has been applied by many EQA providers worldwide. The stability of the EQA material produced by this method can be prolonged for several weeks [8]. Thus, this material can be produced in large volumes for the CBC EQA scheme at a reasonable cost in Vietnam. In the trend of technology, artifacts may be used in adequate control products such as human blood cells, animal blood cells fixed with a crosslinking agent, or hydrogel particles similar in size to human blood cells (diameter and volume) [14,15].

An ideal EQA sample has two essential properties: commutability to allow traceability to a reference system and a target value established with a reference method. If either of these two criteria are not fully met, the between-method variations shown in the EQA program cannot be evaluated [16,17]. Commutable EQA materials have the same inter-assay relationships to clinical samples [12,18,19,20], i.e., they behave like a native patient sample and they have the same mathematical relationship seen in the native clinical samples measured using the observed methods [16,21,22]. However, the large volume of EQA assay material, prepared by pooling ABO-compatible blood components and partially fixed with aldehyde, is noncommutable across different analyser platforms, especially in terms of MCV, WBC, and PLT [8,23]. Possible reasons for this noncommutability could be changes in the cell membranes after the fixation process, the use of nonhuman additives, the pooling of material from multiple donors (resulting in the dilution of sample-specific effects), and manipulating the levels of constituents to obtain abnormal results [21]. Noncommutability leads to matrix-related bias and rises deviation from the assigned value for performance evaluation [16]. Additionally, the provision of traceable EQA assay materials is challenging due to the shortage of reference materials and reference methods for automated cell counters [8,21]. At the time of writing, there is only one appropriate certified reference material (CRM) for haemoglobin measurement (BCR-522, haemoglobin cyanide) and only one accredited reference method in automatic cell counting, the flow cytometric method for platelet counting, listed by the Joint Committee on Traceability in Laboratory Medicine (JCTLM) [8,24,25]. Hence, in this study, we evaluated participants’ results based on the most common procedure used to assign a target value of noncommutable EQA material by categorising participant methods into peer groups that represent similar technologies for performance analysis, calculating the mean or median of the peer-group after the removal of outlier values, and using this as the assigned value [8,12,16].

Performance evaluation based on peer groups from Table 5 showed that there was a tendency to increase the CV (%) as the concentration level decreased for most of the parameters, except MPV. In particular, this relationship can be noticed in platelet count (PLT) as the all-methods CV value declined from about 5.2% at level 1 to about 2.0% at level 3. Although variation was seen for different analysers, the CV was inversely proportional to the platelet count for all analyser models. This trend can also be seen in the study of De la Salle B [8], who evaluated 29 EQA specimen pools with PLT between 5 and 64 × 10^9^/L, distributed to more than 1100 users of 23 different haematology analyser models. The CV value ranged from an all-methods value of about 32% at platelet counts of 5 to 10 × 10^9^/L to about 10% at counts of 36 to 65 × 10^9^/L [26]. Furthermore, the CV range (3-8%) was enlarged with the highest CV value (7.7 %) at a low platelet count (level 1, 94.6–122.4 × 10^9^/L) in this study.

Similarly, De la Salle B [8] also reported the CV range of 15–43% at counts between 5 and 10 × 10^9^/L for all automatic counters, demonstrating that reduced precision between analysers occurs with thrombocytopenic specimens. It is reasonable to assume that the platelet size and morphological changes, such red cell schistocytes, leucocyte fragments, and lipid or protein aggregates are the two main factors that cause automated platelet counting methods to obtain higher CVs at level 1 [27]. In addition, the CV ranges (mostly < 3%) of red blood cell parameters for all peer groups tended to be narrower when compared to the CV range of platelets (PLTs and MPV) and white blood cells (WBCs) at both low and high levels.

In any case, different performances between analyser models are due to the nature of noncommutable EQA material and measurement techniques. These factors should be taken into account in analytical performance evaluation for clinical laboratories.

## 5. Conclusions

The stabilised CBC specimens prepared in this study meet the requirements of ISO/IEC 17043:2010 in terms of the homogeneity and long-term stability of 10 parameters for 3 months. Thus, despite the lack of commutability among peer-groups, these samples can be used as control materials for the CBC EQA scheme in Vietnam based on the same methods/instruments for performance evaluation.

## Figures and Tables

**Table 1:** Homogeneity of specimens.

Parameter	Level 1 (n = 10)			Level 2 (n = 10)			Level 3 (n = 10)		
	Mean	s_s_	0.3 σ_pt_	Mean	s_s_	0.3 σ_pt_	Mean	s_s_	0.3 σ_pt_
RBC (10^12^/L)	1.78	0.00	0.03	4.07	0.00	0.07	5.48	0.02	0.10
Hb (g/L)	48.00	0.00	1.01	111.00	0.00	2.33	151.5	0.42	3.18
Hct (%)	13.90	0.02	0.25	32.20	0.05	0.58	44.37	0.19	0.80
MCH (pg)	27.00	0.03	0.73	27.15	0.07	0.73	27.65	0.11	0.75
MCV (fL)	78.10	0.03	2.34	78.70	0.15	2.36	80.97	0.09	2.43
MCHC (g/L)	345.50	0.46	7.26	344.50	0.75	7.23	341.40	0.64	7.17
RDW-CV (%)	14.75	0.03	0.04	14.95	0.02	0.04	14.90	0.03	0.04
WBC (10^9^/L)	2.83	0.01	0.13	6.24	0.09	0.28	29.03	0.09	1.31
PLT (10^9^/L)	43.00	1.13	3.23	207.50	1.25	15.56	698.00	0.17	52.35
MPV (fL)	13.40	0.07	0.52	13.00	0.15	0.51	12.00	0.18	0.47

s_s_: Estimate of difference between sample standard deviation; σ_pt_: standard deviation for proficiency assessment; mean: the average results of each parameter via measurement in duplicate for 10 samples.

**Table 2:** Stability of specimens at three levels.

Level		RBC (T/L)	Hb (g/L)	Hct %	MCH (pg)	MCV (fL)	MCHC (g/L)	RD %	WBC (G/L)	PLT (G/L)	MPV (fL)
1 (n = 30)	$\bar{y}$ _1_	1.78	48.00	13.90	27.00	78.10	345.50	14.75	2.83	43.00	13.40
	$\left|{\bar{y}}_{12}-{\bar{y}}_{1}\right|$	0.00	0.00	0.20	0.10	0.89	5.10	0.35	0.10	1.00	0.00
	$\left|{\bar{y}}_{13}-{\bar{y}}_{1}\right|$	0.00	0.00	0.22	0.10	1.00	5.60	0.45	0.12	2.00	0.10
	$\left|{\bar{y}}_{14}-{\bar{y}}_{1}\right|$	0.02	0.00	0.30	0.35	0.79	7.50	0.65	0.16	2.50	0.10
	$\left|{\bar{y}}_{15}-{\bar{y}}_{1}\right|$	0.03	0.00	0.35	0.45	0.63	8.70	0.85	0.20	3.50	0.00
	$\left|{\bar{y}}_{16}-{\bar{y}}_{1}\right|$	0.01	0.25	0.40	0.10	1.57	8.10	1.40	0.26	7.00	0.10
	0.3 σ_pt_	0.03	1.01	0.25	0.73	2.34	7.26	0.44	0.13	3.23	0.52
2 (n = 30)	$\bar{y}$ _1_	4.07	111.00	32.20	27.15	78.70	344.50	14.95	6.24	207.50	13.00
	$\left|{\bar{y}}_{12}-{\bar{y}}_{1}\right|$	0.02	0.00	0.24	0.26	1.40	2.30	0.40	0.09	12.50	0.15
	$\left|{\bar{y}}_{13}-{\bar{y}}_{1}\right|$	0.01	0.00	0.40	0.19	1.60	4.00	0.70	0.21	11.50	0.30
	$\left|{\bar{y}}_{14}-{\bar{y}}_{1}\right|$	0.01	0.00	0.61	0.22	2.20	6.20	0.75	0.29	10.00	0.40
	$\left|{\bar{y}}_{15}-{\bar{y}}_{1}\right|$	0.02	0.00	0.56	0.29	2.30	5.70	0.85	0.35	17.50	0.50
	$\left|{\bar{y}}_{16}-{\bar{y}}_{1}\right|$	0.02	0.00	0.63	0.26	2.35	6.40	0.85	0.35	19.50	0.40
	0.3 σ_pt_	0.07	2.33	0.58	0.73	2.36	7.23	0.45	0.28	15.56	0.51
3 (n = 30)	$\bar{y}$ _1_	5.48	151.50	44.37	27.65	80.97	341.40	14.90	29.03	698.00	12.00
	$\left|{\bar{y}}_{12}-{\bar{y}}_{1}\right|$	0.03	3.00	0.78	0.70	1.87	0.80	0.40	0.43	18.00	0.20
	$\left|{\bar{y}}_{13}-{\bar{y}}_{1}\right|$	0.06	2.50	0.98	0.10	0.89	1.80	0.50	0.48	23.00	0.40
	$\left|{\bar{y}}_{14}-{\bar{y}}_{1}\right|$	0.06	2.50	1.13	0.10	1.16	2.90	0.60	1.33	35.00	0.40
	$\left|{\bar{y}}_{15}-{\bar{y}}_{1}\right|$	0.07	2.50	1.08	0.10	0.92	2.60	0.80	1.48	53.00	0.20
	$\left|{\bar{y}}_{16}-{\bar{y}}_{1}\right|$	0.05	3.00	1.23	0.28	1.51	2.65	0.95	1.44	64.75	0.23
	0.3 σ_pt_	0.10	3.18	0.80	0.75	2.43	7.17	0.45	1.31	52.35	0.47

σpt: Standard deviation for proficiency assessment; $\bar{y}$_1_: mean of double measurements right after bottling samples; $\bar{y}$_12_ $,\bar{y}$_13,_ $\bar{y}$_14,_ $\bar{y}$_15,_ $\bar{y}$_16_: the mean of duplicate measurements at 12th, 13th, 14th, 15th, and 16th week of storage; numbers in bold: unstable values at time of storage.

**Table 3:** Evaluation blood cells of CBC EQA material by instrument groups.

Level	Level 1 (N = 78)									Level 3 (N = 75)								
Parameter	RBC (10^12^/L)			PLT (10^9^/L)			WBC (10^9^/L)			RBC (10^12^/L)			PLT (10^9^/L)			WBC (10^9^/L)		
Method: Impedance count	Mean	Um	0.3σ _pt	Mean	Um	0.3σ _pt	Mean	Um	0.3σ _pt	Mean	Um	0.3σ _pt	Mean	Um	0.3σ _pt	Mean	Um	0.3σ _pt
Sysmex XN Series (n = 15)	2.7	0.01	0.05	103.0	1.56	7.73				4.9	0.03	0.09	559.1	4.26	41.94			
Beckman Coulter DxH 600/800/900 series (n = 15)	2.7	0.01	0.05	111.1	2.08	8.33	2.4	0.02	0.11	4.8	0.02	0.09	569.0	5.55	42.68	10.3	0.07	0.46
Mindray BC 1000/2000/3000 series (n = 12)	2.7	0.03	0.05	114.4	1.59	8.58	2.6	0.02	0.12	4.9	0.02	0.09	536.8	3.15	40.26	11.0	0.08	0.49
Nihon Kohden Celtac Alpha (n = 13)	2.7	0.02	0.05	117.9	3.61	8.84	2.6	0.04	0.12	4.8	0.05	0.09	563.7	4.37	42.28	10.0	0.13	0.45
Method: Optical count																		
Siemens/Bayer Advia 120/2120 (n = 13)	2.5	0.02	0.04	94.6	2.02	7.10	2.2	0.03	0.10	4.7	0.04	0.08	526.7	3.35	39.50	9.5	0.10	0.43
Abbott Cell-Dyn Ruby (n = 15)	2.7	0.02	0.05	122.4	2.58	9.18	2.3	0.03	0.10	5.0	0.02	0.09	550.1	4.55	41.26	10.0	0.11	0.45
Sysmex XN Series (n = 15)							2.3	0.02	0.10							10.0	0.08	0.45

**Table 4:** Evaluation RBCs, PLT parameters of CBC EQA material by instrument groups.

Level	Level 1 (N = 78)																				
Parameter	Hb (g/L)			Hct (%)			MCH (pg)			MCHC (g/L)			MCV (fL)			RDW-CV (%)			MPV (fL)		
Manufacturer/Model	Mean	Um	0.3σ _pt	Mean	Um	0.3σ _pt	Mean	Um	0.3σ _pt	Mean	Um	0.3σ _pt	Mean	Um	0.3σ _pt	Mean	Um	0.3σ _pt	Mean	Um	0.3σ _pt
Sysmex XN Series (n = 15)	77.1	0.28	1.62	21.2	0.15	0.38	28.7	0.13	0.77	363.7	2.30	7.64	78.9	0.30	2.37	13.2	0.05	0.04	11.1	0.05	0.43
Abbott Cell-Dyn Ruby (n = 15)	76.6	0.26	1.61	18.1	0.16	0.33	28.5	0.19	0.77	422.4	2.97	8.87	67.5	0.50	2.03	6.9	0.06	0.02	6.4	0.15	0.25
Siemens/Bayer Advia 120/2120 (n = 13)	80.0	0.32	1.68	18.0	0.12	0.32	32.6	0.22	0.88	446.6	3.27	9.38	73.1	0.41	2.19	14.2	0.06	0.04	10.2	0.11	0.40
Beckman Coulter DxH 600/800/900 series (n = 15)	76.5	0.51	1.61	21.7	0.10	0.39	28.6	0.18	0.77	352.9	2.47	7.41	80.9	0.29	2.43	14.5	0.08	0.04	8.5	0.11	0.33
Mindray BC 1000/2000/3000 series (n = 12)	76.6	0.42	1.61	21.7	0.23	0.39	28.2	0.28	0.76	350.6	5.01	7.36	79.8	0.85	2.39	13.4	0.11	0.04	8.9	0.12	0.35
Nihon Kohden Celtac Alpha (n = 13)	78.9	0.93	1.66	21.9	0.27	0.39	29.3	0.21	0.79	359.6	3.01	7.55	81.6	0.68	2.45	14.3	0.27	0.04	8.1	0.24	0.32
**Level**	**Level 3 (N = 75)**																				
Parameter	Hb (g/L)			Hct (%)			MCH (pg)			MCHC (g/L)			MCV (fL)			RDW-CV (%)			MPV (fL)		
Manufacturer/Model	Mean	Um	0.3σ _pt	Mean	Um	0.3σ _pt	Mean	Um	0.3σ _pt	Mean	Um	0.3σ _pt	Mean	Um	0.3σ _pt	Mean	Um	0.3σ _pt	Mean	Um	0.3σ _pt
Sysmex XN Series (n = 15)	139.9	0.35	2.94	39.1	0.21	0.70	28.4	0.17	0.77	358.3	1.77	7.53	79.1	0.27	2.37	13.4	0.09	0.04	11.2	0.07	0.44
Abbott Cell-Dyn Ruby (n = 15)	138.5	0.39	2.91	33.9	0.16	0.61	27.8	0.15	0.75	408.1	1.62	8.57	68.1	0.24	2.04	7.2	0.06	0.02	5.8	0.15	0.23
Siemens/Bayer Advia 120/2120 (n = 13)	141.5	0.34	2.97	33.7	0.33	0.61	30.3	0.23	0.82	419.1	3.78	8.80	72.6	0.60	2.18	12.7	0.11	0.04	10.5	0.09	0.41
Beckman Coulter D × H 600/800/900 series (n = 15)	137.4	0.43	2.89	39.3	0.15	0.71	28.4	0.13	0.77	349.6	1.43	7.34	81.4	0.12	2.44	14.7	0.06	0.04	9.1	0.12	0.36
Mindray BC 1000/2000/3000 series (n = 12)	141.3	0.44	2.97	39.9	0.14	0.72	28.6	0.08	0.77	354.2	1.02	7.44	80.8	0.09	2.42	13.5	0.05	0.04	9.0	0.07	0.35
Nihon Kohden Celtac Alpha (n = 13)	140.8	0.25	2.96	39.0	0.41	0.70	29.2	0.30	0.79	361.0	3.65	7.58	80.9	0.07	2.43	13.8	0.09	0.04	8.4	0.27	0.33

N: Number of results analysed after removal of outliers; n: number of each automated cell counter model; mean: the average results used as the assigned value of the peer groups; CV (%): the coefficient of variation; Um: the standard uncertainty of the assigned value; σ_pt_: the standard deviation for proficiency assessment.

**Table 5:** CV mean of each instrument group for CBC EQA material.

Level	Level 1 (N = 78)									
Parameter	RBC (10^12^/L)	Hb (g/L)	Hct (%)	MCH (pg)	MCHC (g/L)	MCV (fL)	RDW-CV (%)	WBC (10^9^/L)	PLT (10^9^/L)	MPV (fL)
All methods CV (%)	1.7	1.5	2.2	1.8	2.2	1.7	2.1	2.8	5f.2	4.1
Mean CV(%) by instrument										
Sysmex XN Series (n = 15)	1.5	1.1	2.1	1.4	1.9	1.1	1.1	3.0	4.5	1.5
Abbott Cell-Dyn Ruby (n = 15)	2.0	0.9	2.3	1.7	1.8	1.9	2.3	2.9	5.3	6.1
Siemens/Bayer Advia 120/2120 (n = 13)	1.6	1.0	1.7	1.7	1.9	1.4	1.1	2.9	5.4	2.8
Beckman Coulter DxH 600/800/900 series (n = 15)	1.0	1.7	1.2	1.6	1.8	0.9	1.4	2.0	4.7	3.2
Mindray BC 1000/2000/3000 series (n = 12)	2.4	1.4	2.7	2.5	3.6	2.7	2.1	2.0	3.5	3.3
Nihon Kohden Celtac Alpha (n = 13)	1.9	3.0	3.1	1.9	2.1	2.1	4.8	4.2	7.7	7.6
**Level**	**Level 3 (N = 75)**									
Parameter	RBC (10^12^/L)	Hb (g/L)	Hct (%)	MCH (pg)	MCHC (g/L)	MCV (fL)	RDW-CV (%)	WBC (10^9^/L)	PLT (10^9^/L)	MPV (fL)
All methods CV (%)	1.6	0.7	1.7	1.6	1.5	0.8	1.7	2.5	2.0	4.2
Mean CV (%) by instrument										
Sysmex XN Series (n = 15)	1.7	0.8	1.7	1.9	1.5	1.0	2.1	2.4	2.4	2.0
Abbott Cell-Dyn Ruby (n = 15)	1.1	0.7	1.2	1.4	1.1	0.9	2.3	3.0	2.2	6.9
Siemens/Bayer Advia 120/2120 (n = 13)	2.2	0.6	2.4	1.9	2.3	2.1	2.2	2.5	1.6	2.2
Beckman Coulter DxH 600/800/900 series (n = 15)	0.8	0.8	1.0	1.1	1.0	0.4	1.1	1.8	2.5	3.4
Mindray BC 1000/2000/3000 series (n = 12)	1.1	0.9	1.0	0.8	0.8	0.3	0.7	2.1	1.6	2.3
Nihon Kohden Celtac Alpha (n = 13)	2.6	0.4	2.6	2.6	2.6	0.2	1.7	3.3	2.0	8.3

CV: Coefficient of variation; N: number of results analysed after removing outliers; n: number of each automated cell counter model.

## Data Availability

Data is contained within the article.
